# Psychotropic medication prescribing to children and young people mental health inpatient services: An English national study

**DOI:** 10.1177/02698811261473383

**Published:** 2026-08-12

**Authors:** David Branford, Anne Webster, Marinos Kyriakopoulos, Shermin Imran, Prathiba Chitsabesan, Tina Irani, Teresa Randon, Kirsten Peebles, Rohit Shankar

**Affiliations:** 1 Peninsula School of Medicine, University of Plymouth, Truro, UK; 2 NHS England, Redditch, UK; 3 King’s College, London, UK; 4 South London and Maudsley NHS Foundation Trust, Manchester, UK; 5 Greater Manchester Mental Health NHS Foundation Trust, UK; 6 Pennine Care NHS Foundation Trust, Manchester, UK; 7 University College, London, UK; 8 Manchester Metropolitan University, UK; 9 Southern Health, Southampton, UK; 10 Cornwall Partnership NHS Foundation Trust, Truro, UK

**Keywords:** child and adolescent, psychotropic, mental health, pharmacology, polypharmacy, medication

## Abstract

**Background:**

Mental disorders frequently begin in childhood or adolescence. Worldwide, psychotropics are commonly prescribed for under-18s to stabilise behaviour, mood or thoughts. Psychotropics could cause long-term effects on children and young people (CYP) health, growth and development. There is a knowledge gap about psychotropic prescribing in CYP mental health inpatient units.

**Aims:**

To understand and compare psychotropic prescribing patterns in England mental health inpatient units for CYPs and to solicit CYP and their parents’ and carers’ views of it.

**Method:**

A national Quality Improvement programme authorised by National Health Service (NHS) England invited all English CYP mental health inpatient units to participate. The programme included an anonymous medication census to capture psychotropic prescribing practices of participating units. A further two questionnaires sought views of inpatient CYP and their parent carers on the prescribing. All material was co-produced with patient and clinical stakeholders. Quantitative data were presented descriptively and free text responses thematically analysed.

**Results:**

Forty-eight providers overseeing 73 units, that is, 79% of all England NHS commissioned units, with 625 CYP inpatients responded. Of these, 545 (87%) were receiving psychotropics, with 354 (57%) prescribed two or more. Pro re nata (PRN) prescribing practice was variable. Questionnaires were completed by 142 CYP and 75 parent carers. Free text response analysis showed seven themes emerge from the CYP questionnaire and 11 from the parent-carer one.

**Conclusions:**

This is the first English study to systematically explore psychotropic prescribing patterns in CYP mental health inpatient units. The study showed psychotropics are widely prescribed, on a regular and PRN basis.

## Introduction

The mental health of children and young people (CYP) has been the subject of much research in recent years. In England, a 2023 National Health Service (NHS) Digital survey showed probable mental disorder in a fifth of children aged 8–16 years (20.3%) and nearly a quarter of young people aged 17–19 years (23%; NHS Digital, 2023).

### Psychotropic prescribing patterns in CYP

A large General Practitioner (GP) based database, Clinical Practice Research Datalink Aurum database (which covers 21.35% of all GP practices in the UK), was interrogated for antipsychotic prevalence in CYP aged 3–18 years registered in the database in England between 1 January 2000 and 31 December 2019. It showed the period prevalence of all antipsychotic prescriptions increased by an average of 3.3% per year (2.2%–4.9%) and the rate of first prescriptions increased by 2.2% per year (1.7%–2.7%; Radojčić et al., 2023). The authors postulated that the most likely indications of the first identified antipsychotic prescriptions were for autism spectrum disorder, non-affective psychosis, anxiety disorders, depression and conduct disorders. They also argued that there was a trend towards longer treatments. In the USA, a six-fold increase in antipsychotic prescription was noted between 1999 and 2002, with about a third (32%) being prescribed for mood disorders (Olfson et al., 2006). **A continued increase was noted between** 2006 and 2010 for adolescents and young adults but not **for children** aged 12 years or younger (Olfson et al., 2014).

A primary-care database study explored antidepressant prescribing for CYP (5–17 years) across more than 1500 UK general practices between 1998 and 2017 (Jack et al., 2020). The number of 12- to 17-year-olds in England prescribed antidepressants had risen sharply since 2005, with around two-thirds being female (Jack et al., 2020). Antidepressant prescribing rates more than doubled in 12- to 17-year-olds between 2005 and 2017 to 9.7 (females) and 4.2 (males) per 1000 person-years. Another primary-care data study from a population of 1.9 million from Wales, UK, showed antidepressant prescribing increased significantly between 2003 and 2013 (incidence rate ratio (IRR): 1.28), with the increase being mainly in older adolescents (John et al., 2016). The prescription rate was higher in females (IRR: 2.72, 95% CI: 2.60–2.85) and highest in the 15–18 years age group.

The number of children prescribed the hypnotic melatonin increased by 170% in 7 years (The Pharmaceutical Journal, 2022).

### Psychotropic prescribing concerns in CYP

Much of the concern about prescribing of psychotropics has focussed on antipsychotic side effects. Weight gain with second-generation antipsychotics is thought to be greater in CYP than in adults (Correll and Carlson, 2006). A systematic review of 36 studies of CYP with severe mental illness highlighted the vulnerability of CYP admitted to inpatient units and raised concerns of metabolic risk (Carney et al., 2021). These included elevated blood cholesterol, presence of physical health conditions, and behavioural risk factors such as physical inactivity. The review emphasised the need to efficiently monitor, treat and intervene for both physical and mental health.

The NHS Long Term Plan (2017) and the NHS Mental Health implementation plan (2019) set out the priorities for expanding CYP mental health services. This included a psychotropic medication quality improvement (QI) programme sponsored by NHS England for commissioned providers of CYP mental health inpatient services (National Institute for Health and Care Excellence, 2019). The QI programme was designed to enable an insight into the prescribing of psychotropic medications and services and to benchmark individual service prescribing against their peers.

Project aims include

1. To develop effective online data capturing tools that measure current psychotropic medication prescribing practice and the opinions of CYP and parent carers about the medication practice.
2. To provide an overview of medication practices in NHS England commissioned CYP mental health inpatient services with a focus on additional ‘as required: pro re nata’ dosing of psychotropic medications (‘PRN’) and multiple psychotropic medication prescribing (polypharmacy).
3. To give each CYP within the CYP mental health inpatient service and their parent carers the opportunity to evaluate the use of psychotropic medication in their unit and share how they feel about the medication and information provided.
4. To enable CYP mental health inpatient services to compare their psychotropic medication practice with others.

Leading this piece of work throughout the programme was a team representing nursing, pharmacy, autistic young people, parents and project management. All members were involved in all aspects of the work, and the bringing together of diverse experiences, backgrounds and quite differing views at times was beneficial to the project design, delivery and results.

## Methods

The study team worked with providers, clinicians, CYP and parents/carers, listening to their experiences when designing and testing the census tool and questionnaires (Supplemental information 1–3).

### Ethics and governance (Supplemental information 5–7)

This study did not require formal ethical approval as it was considered a QI project (Supplemental information 5). NHS England oversaw the project governance (Supplemental 6). Extensive review and agreement by appropriate NHS England committees to ensure the QI programme met the highest standards in ensuring the rights and privacy of all participants and appropriate steps to protect the confidentiality of information disclosed during the discussions were undertaken (Supplemental information 7). Only the participating unit retained the identity of the individual. Each individual unit was sent a unique code to enable them to access the computer programme and enter the data. The unit entering the data was the only place that had information about who the entries related to. All entries were coded so that nobody outside the unit was aware of the identity of any individual. Each unit was also sent posters with embedded QR codes that solely related to that unit. Parent carers and patients could access the relevant questionnaire via their smartphone. Questionnaires did not ask for any demographic information that could enable anybody to identify them. All entries were coded so that nobody was aware of the identity of any individual. Only de-identified data was submitted to the central dataset. The transfer followed the General Data Protection Regulation (GDPR).

### Census measures (Supplemental information 1)

Versions of the medication census and questionnaires were developed by the authors together with Formic Solutions (an IT company) and piloted by a range of different inpatient providers including general admission units, eating disorder units (ED), psychiatric intensive care units (PICUs) and low secure units.

Completion of Part 1 of the medication census provided information about the mental health unit. Completion of Part 2 provided both demographic information and information about the medication prescribed for the individual. Psychotropic medications included antipsychotics, antidepressants, benzodiazepines and antihistamines used for the management of anxiety, sleep, and for rapid tranquillisation; hypnotics; stimulants used to treat attention deficit hyperactivity disorder (ADHD); antiseizure medications (ASMs) and other psychotropic medication such as lithium. Antimuscarinic medications were included in the scope of the project as they are sometimes used to manage the side effects of the antipsychotics and can impact on cognition. Antihistamines (predominantly promethazine) were used as general sedatives/anxiolytics, in rapid tranquillisation and as hypnotics. Their use was integrated into the parts of the questionnaire relating to the use for general sedation/anxiolytics and to hypnotics.

The programme was designed so that upon provision of the details of the medication, prescribers were given immediate feedback about their prescribing and asked a series of additional questions relating to the extent of psychotropic medication prescribing, the use of PRN medication and multiple psychotropic prescribing (polypharmacy).

Multiple psychotropic medication prescribing (polypharmacy) was categorised as follows:

- Same-Class Polypharmacy – the use of more than one medication from the same class.
- Multi-Class Polypharmacy – the use of more than one psychotropic medication.

### Questionnaire measures (Supplemental information 2 and 3)

Each of the two questionnaires provided questions about various aspects of medication use encompassing both quantitative and qualitative questions (the children and young person questionnaire had 11 questions, the parent-carer questionnaire had 12). They sought information about how well informed and consulted they were about the medications; how helpful they found them and their concerns about the medications.

The questions asked about the same themes so the answers could be jointly analysed and compared. Both questionnaires were only available to complete electronically; they included closed and open questions, and an introduction about how the findings would be used. The qualitative survey data from the online questionnaires were thematically analysed. Narrative responses to the open-ended questions were manually counted, reviewed, explored and coded. The codes were then reviewed and combined into themes. Direct quotes were identified during the process and used in reporting to illustrate particular experiences or themes. Following analysis, relevant responses from CYP and parent carers were compared for the qualitative findings’ summary and overview.

### Recruitment

Commissioned inpatient units in England were identified via NHS England and invited to take part. Responses were reviewed weekly, and regions with minimal response were targeted with personal communication.

Upon registration, participating units were issued with a unique code(s), a technical guidance document and a project guide. In addition, the project team provided weekly drop-in sessions so that participants could respond to any technical problems. Feedback from Formic Solutions enabled the project team to oversee the completion of data and follow up with providers where any data gathering errors or failure to complete were noted. Individual units coded all data, and the questionnaires were completed directly via smartphone technology, maintaining confidentiality.

Completion of the medication census took place between August and November 2022. Data collection for the questionnaires continued throughout a 3-month period. Participants were invited to identify a census date during a 2-week period in mid-September 2022. On the census date, the medication prescribed and the number of doses of PRN medication administered in the previous fortnight were recorded electronically. At the end of the data gathering period, participants were provided with dashboards that provided the following.

1. Extent of their psychotropic medication prescribing, the PRN prescribing, and of polypharmacy practice.
2. The prescribing of their service compared to other mental health services with a similar population and function and compared to all other CYP inpatient services who participated.
3. The views of CYP and parent carers about the prescribing in the service and the medication information and support they receive.

Open-ended survey responses were extracted into an Excel dataset and analysed thematically using an inductive approach informed by Braun and Clarke’s methodology. Consistent with Popping’s classification of ‘really open-ended questions’, responses invited a wide range of spontaneous answers. One researcher manually reviewed, coded and clustered responses into themes by identifying recurring issues, experiences and observations. Direct quotes were selected to illustrate key themes, and findings from CYP were compared with those from parents to produce the qualitative summary and overview.

## Results

### Part A: Medication census

Seventy-nine percent of commissioned mental health inpatient providers for CYP known to NHS England signed up to participate in this QI programme. The most common reason for non-participation of the 21% was staff shortages following COVID.

From those providers (including both NHS and independent sector), census details of the psychotropic medication prescribed for 625 CYP staying in 73 mental health units were recorded. Details of the unit type and their contribution to study numbers are provided in Table 1. Demographics and diagnoses of the study population (*n* = 625) are provided in Table 2. Of note was that 74% of all participants identified themselves as female and 76% of all participants were ethnically white.

**Table 1.** Participating unit type and contribution to study.

Unit type	Medication census	Medication census %
Eating disorder services	123	19.7
General admission units	381	61
General admission units (learning disabilities)	9	1.4
Low secure units	32	5.1
Low secure units (learning disabilities)	10	1.6
Medium secure units	16	2.6
Psychiatric intensive care units	53	8.5
High dependency unit	1	0.2
Calculated totals	625	100

**Table 2.** Demographics and diagnoses of the study population (*n* = 625).

Demographic characteristic/diagnoses	Medication census number	Medication census percentage (%)
Age bands		
12 or younger	27	4
13	33	5
14	74	12
15	138	22
16	155	25
17	174	28
18	13	2
19	5	1
20 or over	6	1
Gender		
Male	144	23
Female	462	74
Other	19	3
Race		
White	475	76
Asian	50	8
Black	50	8
Mixed race	38	6
Other	12	2
Primary diagnoses		
Anxiety	31	5
Attention deficit hyperactivity disorder	21	3
Bipolar disorder	14	2
Depression	82	13
Eating disorder	193	31
First episode psychosis	47	8
Obsessive-compulsive disorder	16	2
Personality disorder/mixed disorder of conduct and emotion/behavioural disorders of childhood	92	15
Post-traumatic stress disorder	58	9
Schizophrenia	35	6
No primary diagnosis	36	6
Autism		
Confirmed diagnosis of autism	124	20
Thought to have autism but not confirmed diagnosis	133	21
No autism	368	59
Detained using mental health act powers		
Detained	431	69
Not detained	194	31
BMI		
Normal BMI	362	58
Low BMI	150	24
High BMI	94	15
Obese	19	3

Of the 625 CYP, 142 (23%) from 39 of the 73 participating units completed the patient questionnaire, and 75 parent carers (12% of the CYP study population) from 32 of the 73 participating units completed their questionnaire. Most units catered for small numbers of CYP, typically 10–12 beds and for high dependency/PICU 3–4 beds.

Some kind of psychotropic medication was prescribed to 545 (87%) CYP. The most widely prescribed were antihistamines and benzodiazepines as sedatives (predominantly as PRN), and antipsychotics, antidepressants, and hypnotics prescribed either as regular (Figure 1) or PRN (Figures 2 and 3).

**Figure 1.**
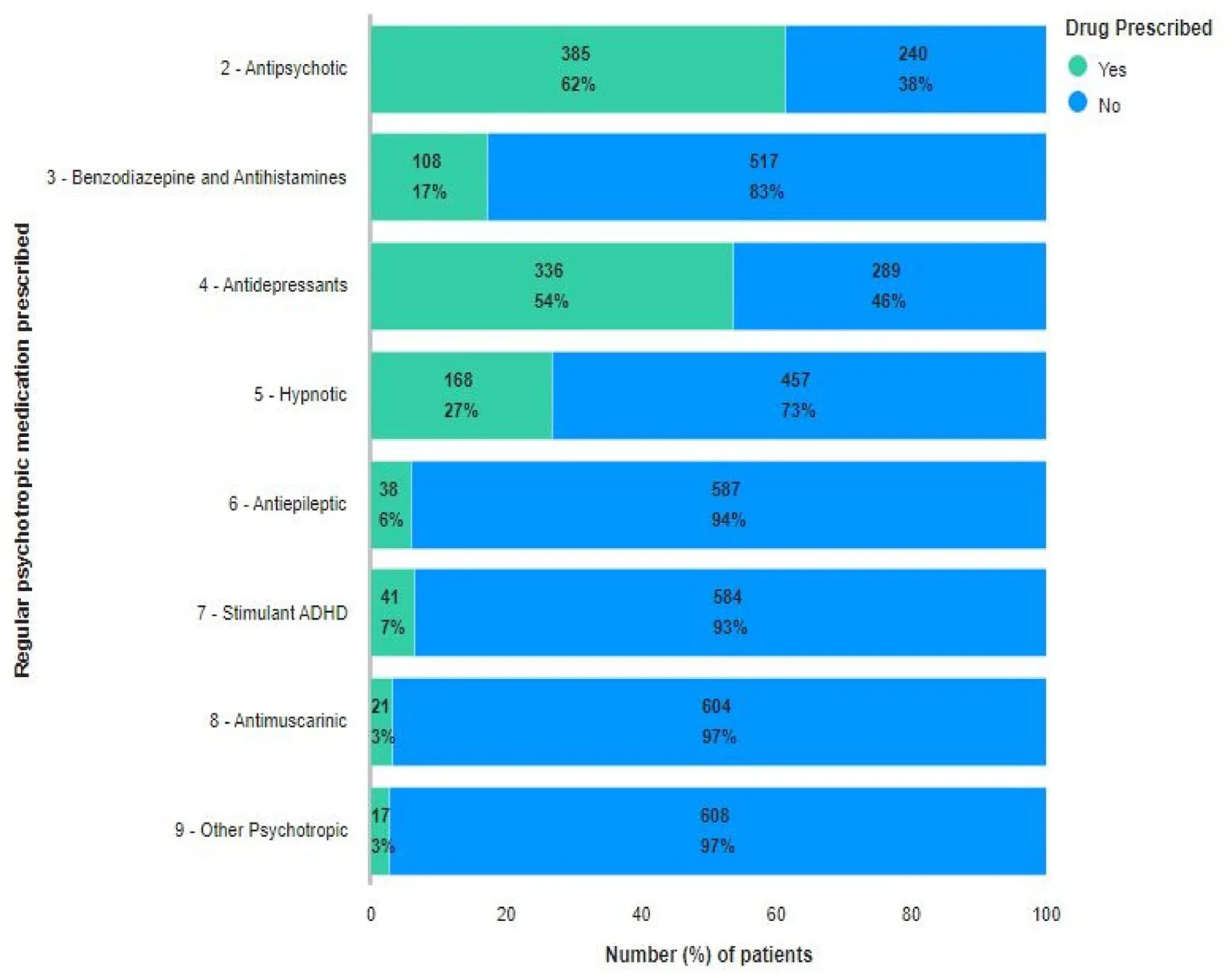
Psychotropic medication categories prescribed regularly.

**Figure 2.**
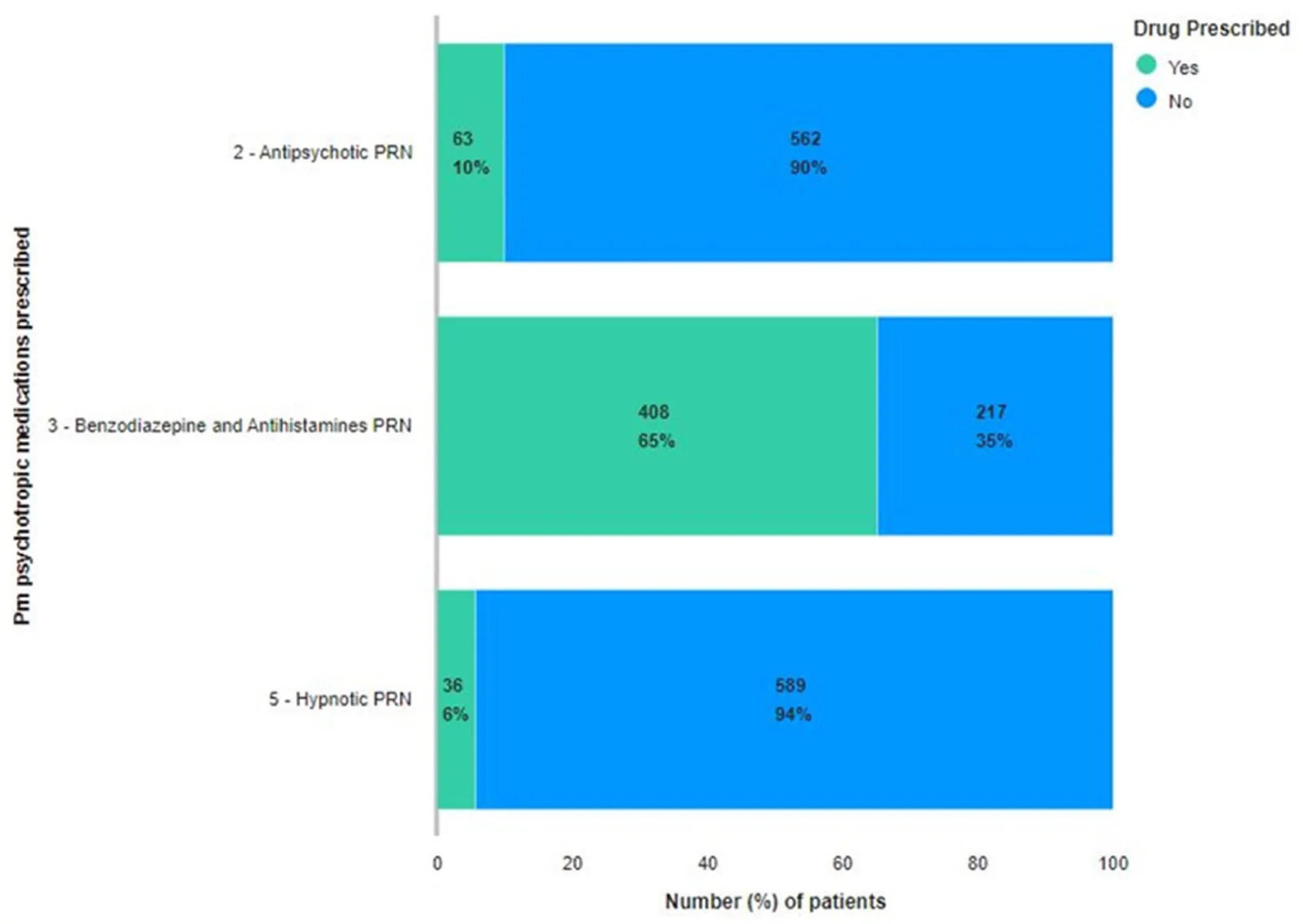
Psychotropic medication categories prescribed as PRN.

**Figure 3.**
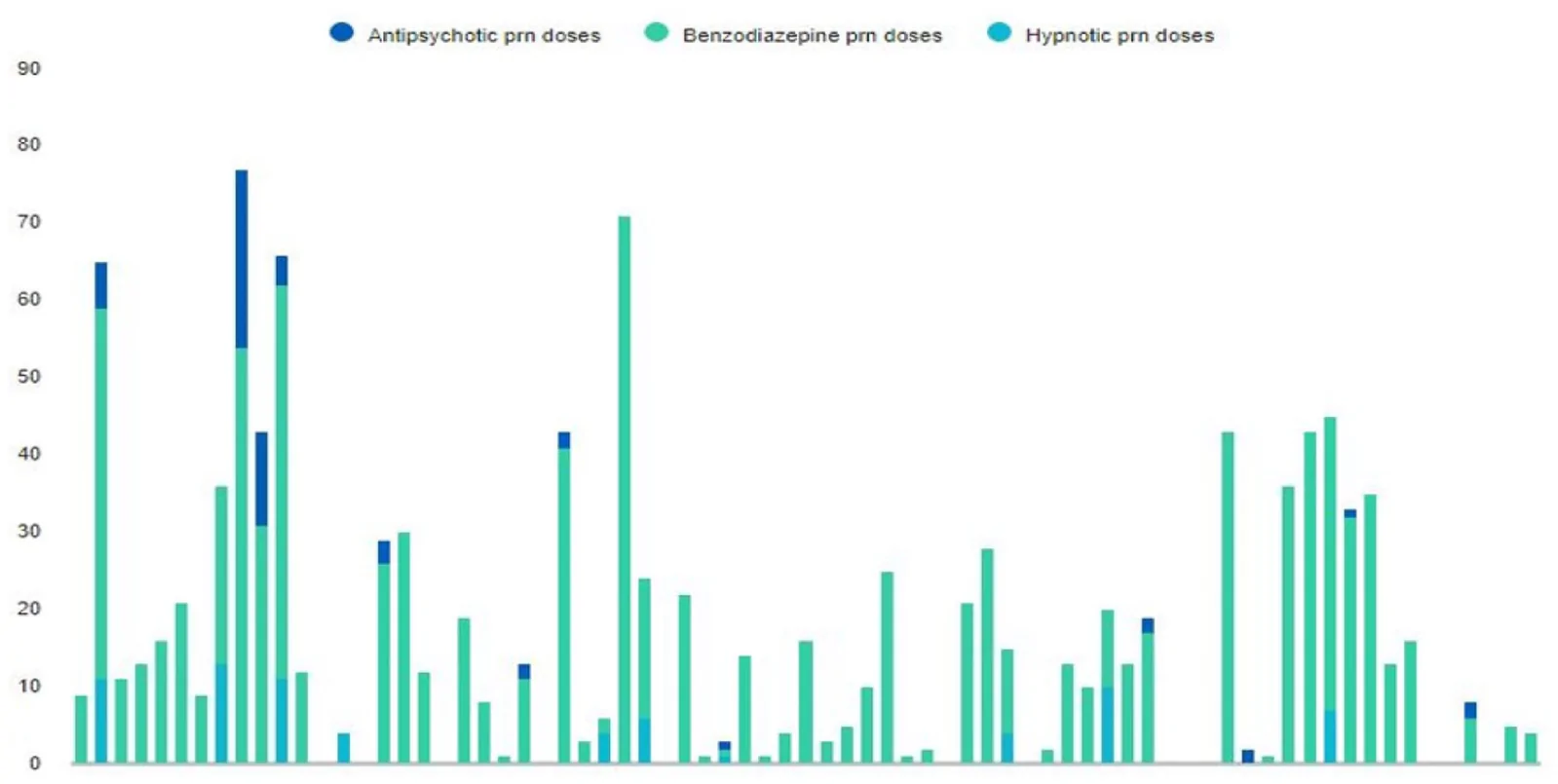
The variation in the number of PRN doses of different psychotropics administered during the 2-week period.

The issue of dose is complex. Most dose ranges quoted in the British National Formulary (BNF) relate to adults, although some have dose ranges for children BNF-C. This makes it difficult to ascertain whether doses are excessive. Three percent of prescribed antipsychotic medication was on doses above adult BNF maximum. Where regular and PRN antipsychotics are both prescribed, 14.2% (9 out of 63) could have combined antipsychotic doses equivalent to above BNF dose maximum when a PRN was given alongside their regular antipsychotic. Prescribers were asked the reasons given for prescribing a regular antipsychotic. They were management of behaviour (24%), mood stabilisation (35%), psychotic phenomena (19%) and other (22%). The other category was dominated by the treatment of anorexia nervosa cognitions of those with EDs. Depression was the most common reason for prescribing an antidepressant (49%), followed by anxiety (35%), and obsessive-compulsive disorder (6%). Antidepressant without a diagnosis of depression or an anxiety disorder (including OCD, etc.) was prescribed to 10% (57 of 545).

Antipsychotics were prescribed to 385 (62%) CYP on a regular basis and for 63 (10%) on a PRN basis. A wide range of antipsychotics was prescribed, the most common being olanzapine (147, (38%)), quetiapine (91, (24%)) and aripiprazole (77, (20%)). The most widely prescribed on a PRN basis were olanzapine (26), haloperidol (16) and levomepromazine (14). Antidepressants were prescribed for 336 (54%) on a regular basis, of which sertraline was the most widely prescribed (163, 49%), followed by fluoxetine (107, 32%). Hypnotics were prescribed to 168 (27%) CYP and 36 (6%) on a PRN basis. The most widely prescribed hypnotic was melatonin. The remaining psychotropic medication categories (ASMs, stimulants for ADHD, antimuscarinics and others such as lithium) were rarely prescribed.

Only 4% of all participants were 12 years or younger. Thus, little inference could be gained on the prescribing in the pre-adolescent sub-group. The prescribing of psychotropic medications generally increased with age and with an increased number of diagnoses other than EDs. Specifically, antipsychotic and hypnotic prescribing increased with age, as did PRN prescribing and polypharmacy. Antidepressant prescribing showed no age pattern. Both the prescribing and administration of PRN psychotropic medications were common. This was an area of high concern for parent carers.

Further enquiry was made about whether autism was diagnosed, thought to be present (but not diagnosed) or absent. More of those diagnosed autistic received antipsychotics (73%) than those thought to be autistic (57%) and those not (60%).

The most widely prescribed PRN medications were the benzodiazepines and antihistamines as anxiolytics/sedatives (408, 65%). Antipsychotics and hypnotics were less frequently prescribed as PRN (10% and 8% respectively). The antihistamine, promethazine (310, 76%), was the most prescribed PRN, followed by benzodiazepines, lorazepam (202, 50%), clonazepam (37, 9%), and diazepam (12, 3%). The most common combination was promethazine and lorazepam, followed by clonazepam and promethazine. The prescribing was primarily to manage behaviour or for rapid tranquillisation. Antihistamines and benzodiazepines were also the most administered PRN medications, either for general management of behaviour or for rapid tranquillisation.

There was a wide variation in the extent to which PRN psychotropic medications were administered in the various units. Four units administered more than 60 doses during the 2-week period prior to the census, while nine units administered none (Figure 3).

Antipsychotics and hypnotics were prescribed and administered less frequently on a PRN basis than antihistamines and benzodiazepines with 21 patients receiving PRN antipsychotics over the 2-week period (maximum number to one CYP was seven times) and 17 patients received a PRN hypnotic (maximum number to one CYP was 11 times).

Both same-class and multiclass polypharmacy were investigated. Same-class polypharmacy was not common; however, multiclass polypharmacy was. More than half CYP (354, 57%) were prescribed more than one regular psychotropic medication (Table 3).

**Table 3.** Number of psychotropic medications prescribed.

Number of psychotropic medications prescribed	Number of CYP (regular psychotropic medications only)	Number of CYP (regular and PRN psychotropic medications)
0	111	81
1	160	72
2	185	104
3	104	129
4	49	116
5	12	69
6	3	30
7	1	19
8	0	3
9	0	2
Total	625	625

### Part B prescriber immediate feedback results

Upon completion of the medication census, prescribers were provided with immediate feedback of their prescribing and the extent of their polypharmacy and asked two questions: the scope to reduce the number of medications and whether they felt there was a strong evidence base to support their prescribing. There were prescriber responses relating to the medication for 438 CYPs (no response relating to 187 CYPs). For 344 (78%) of CYPs, the prescriber felt there was little or no scope to reduce the load (Figure 4), while for the remaining 94 (22%) there was medium or significant scope. Further, over 90% of prescribers fed back that they believed their prescribing of multiple psychotropic medications was supported by an evidence base.

**Figure 4.**
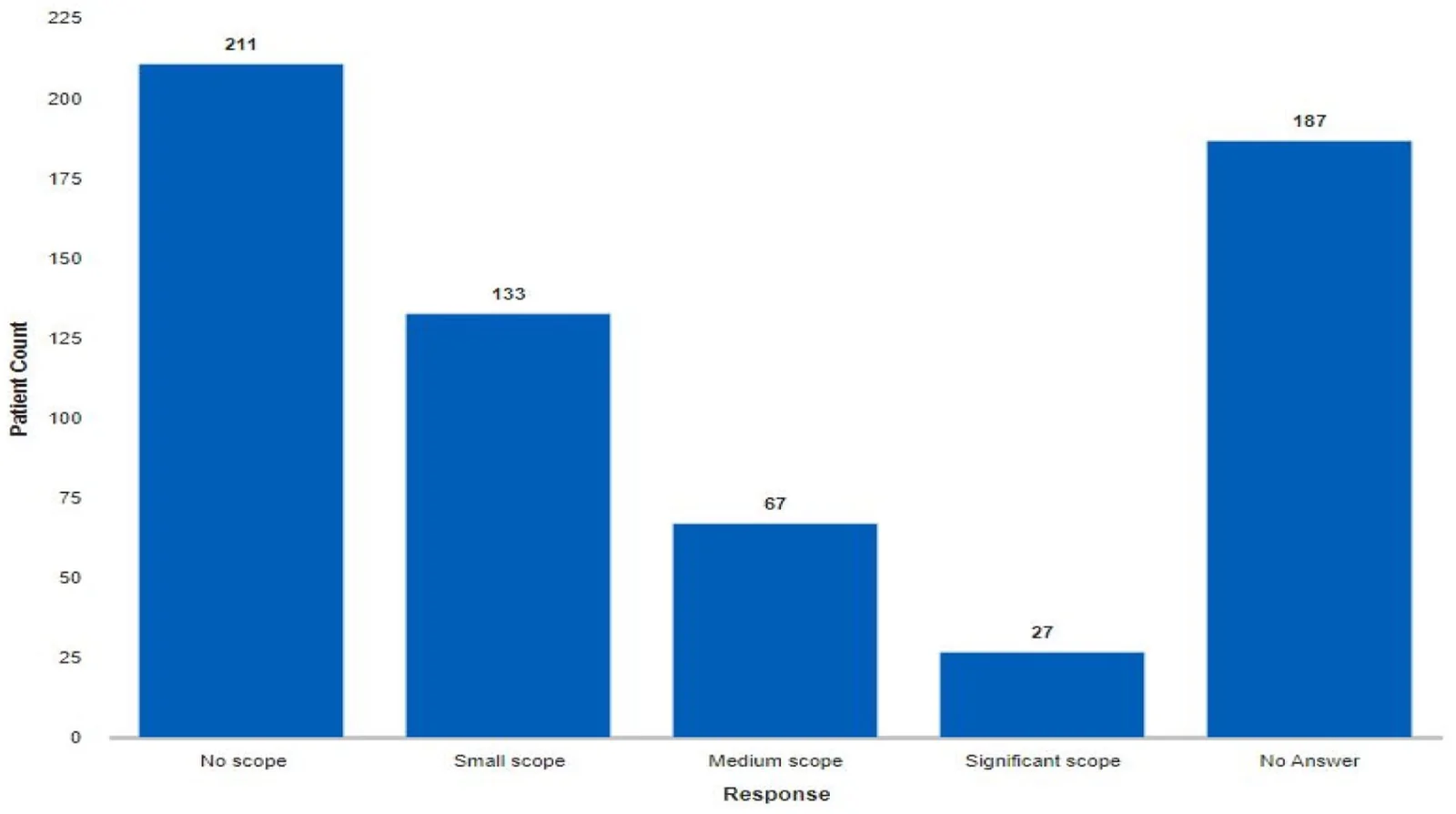
Prescribers’ perception of the potential to reduce the psychotropic medication load.

### CYP and parent/carer questionnaire feedback

Table 4 provides quantitative responses to key questions of the CYP and parent/carer questionnaires. The results of the thematic analysis of the questionnaire open-ended question responses are in Table 5, with each section heading deriving from the question (Table 6).

**Table 4.** Quantitative responses from CYP questionnaire and parent-carer questionnaire.

Question	No of responses CYP questionnaire (%)	No of responses parent-carer questionnaire (%)
- Know what medications are?	Yes. 119 (89)	Know all 59 (83)
	No. 7 (5)	Know some 9 (13)
	Don’t know 8 (6)	No 3 (2)
		Not sure 1 (1)
- Enough information to ask questions?		Yes. 57 (79)
		No. 13 (18)
		Don’t know 2 (3)
- Does medication help?	Yes. 64 (49)	All helps 26 (37)
	No. 24 (18)	No 2 (3)
	Don’t know 44 (33)	Some helps 36 (51)
		Don’t know 7 (10)
- Member of staff to ask questions?	Yes. 111 (82)	Yes. 64 (90)
	No. 12 (9)	No. 7 (10)
	Don’t know 12 (9)	
- Medication stop you doing things/impact on wellbeing?	Yes. 8 (6)	Yes. 8 (6)
	No. 56 (42)	No. 56 (42)
	Sometimes 47 (39)	Sometimes 47 (39)
	Don’t know 23 (17)	Don’t know 23 (17)
- Any worries about the medication	Yes. 42 (31)	Yes. 35(49)
	No. 72 (54)	No. 36 (51)
	Don’t know 20 (15)	
- Feel your views are listened to?		Yes 57 (81)
		No 8 (11)
		Don’t discuss 5 (7)
- Information helpful	Yes. 62 (47)	Yes. 50 (75)
	No. 5 (4)	No. 2 (3)
	Sometimes 49 (37)	Somewhat 12 (18)
	Don’t know 15 (11)	Don’t get 3 (4)

**Table 5.** Thematic analysis of open-ended questions from CYP and parent-carer questionnaires.

Theme CYP questionnaire	Number of open-ended responses	Summary	Theme parent-carer questionnaire	Number of open-ended responses	Summary
Understanding why medication is taken	114	Generally understood that they took medication for their mental health problem/s	Why their child is prescribed medication	71	All understood that their child was being prescribed medication for their mental health problem/s
			Ability to ask informed questions	8	Mixed responses, some parents saying they did not have enough information generally or enough to ask questions about medication
			Staff approached with questions about the child’s medication	49	Most experiences were positive, with two saying that the psychiatrist or nurses had already given them sufficient information so asking questions was not necessary
Ease of asking staff questions about medication	73	Wanted • Greater opportunities to speak directly with the clinician • Staff to be more proactive in asking them questions and to have accurate knowledge about their medication Staff skills and attitudes made a difference	Asking staff questions about the child’s medication	43	Half said they were satisfied, and that they could ask questions about medication easily, citing being ‘confident’, readily able to ask available staff or already well informed
			Perceived helpfulness of the child’s medication	42	Mixed responses. Some parents felt that medication was helping their child with symptom reduction while several parents found medication review and change helped, others thought that medication was not working and were concerned about dosage increases or medication changes Several were unsure if a specific medication was helping or if taking multiple medications, which one was helping
Impact of medication on children and young people doing things they enjoy	27	Half said that the medication made the ‘tired’, ‘sleepy’, ‘drowsy’ or lacking energy, no motivation to do stuff’ 1 quote medication can make me feel a bit like a zombie’	Perceived effect of medication on the child’s capacity to do things they enjoy	28	Over a third specifically reported that sedation and drowsiness seemed to be problematic side effects. Described their children as being ‘dazed’, ‘spaced out’, ‘lethargic’, fuzzy headed’, ‘drugged up’, ‘tired’, ‘drowsy’ or ‘dopey’. Several said that sedative side effects had a negative impact on their child’s motivation as well as their ability to concentrate, undertake basic tasks and participate in activities
Concerns about taking medication	37	Weight gain primary concern. Worried about the long-term effects of taking medication such as addiction, the medication no longer working or that it would ‘make things worse’ in the long run	Concerns about the child’s medication	34	Nearly one-third were worried about the long-term effects of the medication, including dependency, reduced effectiveness and the development of additional side effects over time
Approaches to staying healthy and well	78	Just under half said that exercise, fitness, or sport helped them to stay healthy. They mentioned activities such as going to the gym, cycling, football, horse riding, swimming, and walking. Diet and healthy eating			
Alternative sources of information about medication	14	Nearly half said they consulted online sources for more information, such as Google and the NHS website. One mentioned going to the Young Minds website	Accessing information about the child’s medication	8	Information was given either verbally or in written form
Improving information about medication	19	Needs accessibility, conciseness, and clarity	Helpfulness of information about medication	8	Some parents reported that the information sheets they had received were helpful
			Involvement in decision making about child’s medication	20	Some said that they were not consulted, listened to, or did not feel that their expertise or concerns were taken into account. Others reported being regularly consulted and involved in discussions
			Helpful alternatives to medication	48	Parents described a wide range of alternatives to medication, either referring to what their child was already receiving or to a wish that alternatives were available. The vast majority believed the alternatives were or could be beneficial to their child. Many referred to psychological interventions such as ‘talking therapies’, counselling, psychotherapy, family therapy, cognitive behavioural therapy, and dialectical behavioural therapy

**Table 6.** Recommendations for providers.

Recommendations for providers	
Challenge widespread prescribing	Challenge the widespread prescribing of psychotropic medications, the prescribing and administration of ‘PRN’ psychotropic medications and the prescribing of multiple psychotropic medications
Minimise medications when not beneficial	Offer robust strategies to minimise continued use of psychotropic medications when they are no longer of benefit
Share information	Consider best ways to share information about psychotropic medication and planned prescribing, including use of ‘PRN’ with children, young people, and their parent/carers
Ask, listen, do	Consider opportunities for each child, young person, and their parent/carer to ask questions, weigh up benefits and risks and express their views and concerns in ways that are responsive to their needs, and consider how their views can inform prescribing and treatment
Involve in decision making	Best involve each child, young person and parent/carer in decision making about psychotropic medication use, alternative/additional treatments, and other ways to be healthy

Both CYP and parent carers understood that medication is used to help reduce symptoms, behaviours and feelings associated with mental health problems. While parents felt that medication could help their child, they were concerned about effectiveness and the impact of taking multiple medications.

Medication side effects such as drowsiness and lethargy were of concern to both CYP and parent carers. They said these impact on motivation, engagement and concentration in a way that can stop CYP from doing things they enjoy. CYP were particularly worried about weight gain as a medication side effect. Parent carers were concerned about the longer-term effects of medication, including dependency and physical health, as well as the impact of changing or stopping treatment.

CYP said that healthy eating and exercise help them to stay healthy and well. They also said that activities, hobbies and being creative can help. Parent carers felt that alternatives to medication such as psychological, occupational, and other therapies can be beneficial for their child and want them to be available.

Having clear, concise written information and regular or open opportunities to engage with approachable, knowledgeable staff made it easier for CYP and parents to ask questions about medication. They said that they would also like staff to be proactive.

CYP consulted online sources for information about medication. CYP and parents said they want concise, accessible information on medication that covers side effects and is delivered in non-clinical language. Parent carers asked clinical staff, including nurses and consultant psychiatrists, questions about medication. They were not always involved in decisions about their child’s medication but wanted to be involved and taken seriously.

Although information about the prescribing of regular psychotropic medication was available, parent carers said information about PRN medications, when they were likely to be used and how often they were being used, was not so well communicated.

## Discussion

Nearly four of five NHS England commissioned inpatient units for CYP participated in this national study. About a quarter were from the independent sector.

Both CYP and parent carers were provided with an opportunity to comment on their involvement with the prescribing process, the use of medications and their knowledge of them. It identified CYP being prescribed psychotropic medications largely from five groups: antipsychotics, antidepressants, antihistamines, benzodiazepines and hypnotics. A combination of psychotropic medications (polypharmacy) and the use of PRN psychotropic medication was common.

Our study shows widespread prescribing of psychotropics, with many receiving antipsychotics, benzodiazepines and antihistamines to manage behaviour. A noted lack of evidence base leads to particular concern about its role in facilitating restrictive practices. Use of psychotropic medication for managing behaviour is seen as chemical restraint and can have multiple significant consequences (Muir-Cochrane et al., 2020). This includes overshadowing suitable staffing resources, availability of alternative de-escalation strategies and risk of iatrogenic harm (Danda, 2022; Muir-Cochrane and Oster, 2021).

One striking observation was the prescribing of olanzapine. Of those receiving it, 38% were for EDs. A recent review emphasises the lack of evidence surrounding medication use for anorexia nervosa and that caution must be taken when administering medication, due to the physical complications such as cardiac problems (Resmark et al., 2019). The NICE guidelines also state that there is no proven benefit of combined treatment over psychotherapy alone in treating patients without comorbidities.

Antidepressants were prescribed for more than half (54%) of the CYPs, depression and anxiety being the main reasons for this prescribing. There are no other comparable studies from mental health inpatient facilities for CYP. However, this is a very high rate of prescribing compared to primary-care–based data (Jack et al., 2020; John et al., 2016). For those with moderate to severe depression, NICE recommends more intense psychological therapies (such as individual CBT) as initial treatment. However, the guidelines state that combined therapy (fluoxetine and psychological therapy) is an alternative initial treatment for 12- to 18-year-olds after assessment by a specialist. While the average effect of the majority of the newer antidepressants in reducing depression symptoms is small, depression is a heterogenous condition, and some individuals may experience a greater response (Hetrick et al., 2021).

The antihistamine promethazine was widely prescribed in over three-quarters of CYP (76%) for varying indications, including general sedation, sleep, anxiety and rapid tranquillisation. There is little evidence from clinical trials to support the use of promethazine in these many circumstances. The Maudsley Guidelines (Taylor et al., 2018) list promethazine as a useful sedative as an alternative to antipsychotics; however, it has a slow onset in rapid tranquillisation. There is a need to further explore the reason for the high use of promethazine and whether it is being used as an alternative to benzodiazepines because of concerns about their potential dependence, paradoxical agitation and possible diversion.

Over a quarter (27%) of CYP were receiving hypnotics on a regular basis and a further 6% on a PRN basis. Melatonin was the most widely prescribed. Melatonin taken in low-to-moderate doses (e.g., 5 mg daily or less) for short periods appears safe and well-tolerated (Givler et al., 2023). While there is developing evidence of some benefits of prescribing for neurodevelopmental conditions, generally there is no evidence of its effectiveness or side effects long-term (Givler et al., 2023). Whether CYP were admitted receiving regular melatonin or whether first active sleep hygiene programmes were in place was not investigated.

There was extensive prescribing and administration of PRN psychotropic medications. A wide variation in the use of PRNs is to be expected considering the diverse populations and a range of functions of the units; however, there remained a large variation in the extent of PRN prescribing between similar units. PRN prescribing is a valuable facility, particularly in those with acute, fluctuating mental state and behaviours; however, it is open to misuse and may be unnecessary or inappropriate. It also increases the risk of patients being given higher doses than would usually be recommended. PRN prescribing was an area of high concern for parent/carers. Although information about the prescribing of regular psychotropic medication was available, parent/carers said information about ‘PRN’ medications, when they were likely to be used and how often they were being used, was not so well communicated. A systematic review of PRN psychotropic medicines in long-term care settings concluded that further initiatives are needed to improve PRN medicines management (Vaismoradi et al., 2019). This includes a need to explore factors that affect PRN prescription and administration and to develop appropriate PRN guidelines to prevent harm and improve safety. Our results suggest a greater level of co-operation and standard setting between inpatient services is warranted to streamline practices, given the wide variation in PRN use.

Over half of CYP were prescribed more than one regular psychotropic medication usually multiclass polypharmacy. It was not clear whether this multiclass polypharmacy was aimed at supplementing a partially successful treatment response rather than stopping and initiating alternative treatment, often at a time of instability and crisis for CYP during inpatient admission. It needs to be mentioned that when treating severe depression in a person with schizophrenia, or in the treatment of bipolar disorder where combination therapy is recommended if monotherapy has failed, multi-class polytherapy could be appropriate. The relationship to multiple diagnoses and multi-class prescribing was not investigated.

There was a range of reasons given for the level of polypharmacy and feeling unable to reduce the psychotropic load. These included the shortness of the stay in the unit, the CYP often being admitted on a combination of medications, and it not being seen as a priority for the residential unit. The expectation was that removing any unnecessary medications would occur in the community following discharge. However, the issue of whether that occurred was not investigated. It is worth bearing in mind that inpatient settings generally would be felt to offer better structure, consistency and qualified staff to necessitate any withdrawal than community settings. However, except for the longer stay units such as medium secure units, one is left wondering where, if anywhere, such a reduction would take place, as the units are under great pressure to discharge as soon as possible. This service dilemma risks over-medication if community teams do not feel comfortable reducing medications in a CYP who has been so unwell they have required a hospital admission.

These findings confirmed the parental concerns from the need for shared decision making; many parent carers were unclear why their CYP needed to be on so many and diverse medications.

Further investigation is also needed into strategies to monitor, prevent and respond to potential common side effects of psychotropics such as drowsiness and excessive weight gain. Most antipsychotics are associated with weight gain (Campforts et al., 2023).

The psychotropic prescribing identified in our inpatient cohort reflects wider international trends seen across England and the USA over the past two decades (McLaren et al., 2024). English and US studies have both demonstrated sustained rises in antipsychotic and antidepressant prescribing in CYP, particularly for non-psychotic disorders. This is likely related to greater recognition of mental disorders, increasing numbers of CYP accessing specialist mental health services, and wider acceptance of pharmacological treatment approaches. However, relatively few psychotropic medications are specifically licensed for CYP, meaning much prescribing remains off-label. Our findings therefore align with broader international concerns regarding increasing psychotropic use and polypharmacy in young people.

### Limitations of the programme

The focus of the study was on inpatient units. All community CYP services would have been significantly out of the scope of our study. Inpatient units have the most unwell and complex group of CYP and are generally expected to provide similar services. While community CYP services would be variable based on geography and commissioning.

The census did not ask details of non-medication therapeutic strategies used by prescribers alongside medication. This could potentially impact on use of psychotropics. The need for greater access to all kinds of alternatives to medication was highlighted in the questionnaire feedback.

It was not investigated if the medications were prescribed prior to admission, the extent of new or altered prescribing that occurred during admission, and how long CYP remained on the various medications.

Evidence that the use of medications as described within this QI project led to either favourable or unfavourable outcomes in terms of treatment of mental illness and length of stay was not investigated.

Although the issue of workforce in mental health wards has been highlighted by the Care Quality Commission (CQC, 2019), the census did not ask for specific issues of workforce in the participating units. Informal discussions with professionals during linked webinars alluded to workforce difficulties across units likely to have persisted due to the COVID pandemic. Also, the proximity to the COVID period may have influenced the results. However, this work provides a baseline for future work related to the long-term impacts of COVID on the management of mental health conditions for CYP.

It is also worth noting that a lack of evidence does not mean that the prescribing was likely to be inappropriate, and there could be a more nuanced explanation of the complexity of treating mental illness in young people, especially as this is an area where published evidence is limited.

To ensure confidentiality, the two questionnaires could only be accessed and completed electronically. This may have limited their completion by CYP and their parent carers. Also, there was no way of knowing whether there was duplication of responses or whether persons other than CYPs and their parent carers responded.

We did not collect data on non-pharmacological restrictive practices in the participating units. This prevents us from providing a comparison of whether there were higher non-pharmacological restrictive practices in the units that had low use or did not use prn medication.

## Conclusion

This has been a first opportunity for commissioned providers of CYP mental health inpatient services in England to systematically and anonymously provide, explore and review their patterns of psychotropic medication use compared to their peers. The very high response rate brings confidence to the findings outlined in this study. Although the primary purpose of this programme was for providers to develop their own improvement plans, recommendations for providers were proposed and circulated (Table 5).

### Ethical information (Supplemental information 5–7)

This study did not require formal NHS ethical approval as it was considered a QI project (Supplemental information 5). NHS England oversaw the project governance (Supplemental 6). Extensive review and agreement by appropriate NHS England committees ensured the QI programme met the highest standards in ensuring the rights and privacy of all participants and that appropriate steps to protect the confidentiality of information disclosed during the discussions were undertaken (Supplemental information 7). Only the participating unit retained the identity of the individual. Each individual unit was sent a unique code to enable them to access the computer programme and enter the data. The unit entering the data was the only place that had information about who the entries related to. All entries were coded so that nobody outside the unit was aware of the identity of any individual. Each unit was also sent posters with embedded QR codes that solely related to that unit. Parent carers and patients could access the relevant questionnaire via their smartphone. Questionnaires did not ask for any demographic information that could enable anybody to identify them. All entries were coded so that nobody was aware of the identity of any individual. Only de-identified data was submitted to the central dataset. The transfer followed the GDPR.

## Supplemental Material

sj-docx-1-jop-10.1177_02698811261473383 – Supplemental material for Psychotropic medication prescribing to children and young people mental health inpatient services: An English national study Supplemental material, sj-docx-1-jop-10.1177_02698811261473383 for Psychotropic medication prescribing to children and young people mental health inpatient services: An English national study by David Branford, Anne Webster, Marinos Kyriakopoulos, Shermin Imran, Prathiba Chitsabesan, Tina Irani, Teresa Randon, Kirsten Peebles and Rohit Shankar in Journal of Psychopharmacology

sj-docx-2-jop-10.1177_02698811261473383 – Supplemental material for Psychotropic medication prescribing to children and young people mental health inpatient services: An English national study Supplemental material, sj-docx-2-jop-10.1177_02698811261473383 for Psychotropic medication prescribing to children and young people mental health inpatient services: An English national study by David Branford, Anne Webster, Marinos Kyriakopoulos, Shermin Imran, Prathiba Chitsabesan, Tina Irani, Teresa Randon, Kirsten Peebles and Rohit Shankar in Journal of Psychopharmacology

sj-docx-3-jop-10.1177_02698811261473383 – Supplemental material for Psychotropic medication prescribing to children and young people mental health inpatient services: An English national study Supplemental material, sj-docx-3-jop-10.1177_02698811261473383 for Psychotropic medication prescribing to children and young people mental health inpatient services: An English national study by David Branford, Anne Webster, Marinos Kyriakopoulos, Shermin Imran, Prathiba Chitsabesan, Tina Irani, Teresa Randon, Kirsten Peebles and Rohit Shankar in Journal of Psychopharmacology

sj-docx-4-jop-10.1177_02698811261473383 – Supplemental material for Psychotropic medication prescribing to children and young people mental health inpatient services: An English national study Supplemental material, sj-docx-4-jop-10.1177_02698811261473383 for Psychotropic medication prescribing to children and young people mental health inpatient services: An English national study by David Branford, Anne Webster, Marinos Kyriakopoulos, Shermin Imran, Prathiba Chitsabesan, Tina Irani, Teresa Randon, Kirsten Peebles and Rohit Shankar in Journal of Psychopharmacology

sj-docx-5-jop-10.1177_02698811261473383 – Supplemental material for Psychotropic medication prescribing to children and young people mental health inpatient services: An English national study Supplemental material, sj-docx-5-jop-10.1177_02698811261473383 for Psychotropic medication prescribing to children and young people mental health inpatient services: An English national study by David Branford, Anne Webster, Marinos Kyriakopoulos, Shermin Imran, Prathiba Chitsabesan, Tina Irani, Teresa Randon, Kirsten Peebles and Rohit Shankar in Journal of Psychopharmacology

sj-docx-6-jop-10.1177_02698811261473383 – Supplemental material for Psychotropic medication prescribing to children and young people mental health inpatient services: An English national study Supplemental material, sj-docx-6-jop-10.1177_02698811261473383 for Psychotropic medication prescribing to children and young people mental health inpatient services: An English national study by David Branford, Anne Webster, Marinos Kyriakopoulos, Shermin Imran, Prathiba Chitsabesan, Tina Irani, Teresa Randon, Kirsten Peebles and Rohit Shankar in Journal of Psychopharmacology

sj-docx-7-jop-10.1177_02698811261473383 – Supplemental material for Psychotropic medication prescribing to children and young people mental health inpatient services: An English national study Supplemental material, sj-docx-7-jop-10.1177_02698811261473383 for Psychotropic medication prescribing to children and young people mental health inpatient services: An English national study by David Branford, Anne Webster, Marinos Kyriakopoulos, Shermin Imran, Prathiba Chitsabesan, Tina Irani, Teresa Randon, Kirsten Peebles and Rohit Shankar in Journal of Psychopharmacology

## Data Availability

The data that support the findings of this study are available from the corresponding author upon reasonable request.*
