# Supplementary material for: Psychotropic medication prescribing to children and young people mental health inpatient services: An English national study
Source: J Psychopharmacol. 2026 Aug 12;40(10):1594–607. doi: 10.1177/02698811261473383 (PMC13683887; doi:10.1177/02698811261473383)
Supplement: Supplementary file 2 [file sj-docx-7-jop-10.1177_02698811261473383.docx]

Psychotropic Medication in Children and Young People's Mental Health Inpatient Services Quality Improvement Programme

Child and Young Person Questionnaire

Who is this questionnaire for?

This questionnaire is for any child or young person who is:
a) staying in this inpatient unit, and
b) taking psychotropic medication

There is also a questionnaire for parent carers. Ask the staff if you want to find out more about the parent carer questionnaire.

People will not know it is you who has given your answers to each question

The information you give us will be anonymous.

You won't be asked to give your name, date of birth or address.

Any names of people that are included in people's answers will be deleted before the answers are shared with the unit.

People looking at the answers from this questionnaire will not know which child or young person has given each answer.
This means that people including members of staff from your unit will not be able to see that it is you who has given your answers.

Psychotropic Medication in Children and Young People's Mental Health Inpatient Services Quality Improvement Programme

Child and Young Person Questionnaire

This questionnaire is from NHS England Quality Transformation Team

They are working with inpatient services including the one you are staying in.

They want to make sure that children and young people are getting the right medication and treatment at the right time and for the right reasons.

By answering the questions, you will help them to understand your experiences and how you feel about your medication and support.

What will happen to the information I am giving?

NHS England are asking lots of children and young people to complete this questionnaire.

Everyone's answers will be put together so NHS England and each unit taking part in this piece of work can see what children and young people are saying about medication.

NHS England, this inpatient unit, and other inpatient units in England will use the answers to help improve care and treatment for children and young people.
