# Supplementary material for: Psychotropic medication prescribing to children and young people mental health inpatient services: An English national study
Source: J Psychopharmacol. 2026 Aug 12;40(10):1594–607. doi: 10.1177/02698811261473383 (PMC13683887; doi:10.1177/02698811261473383)
Supplement: Supplementary file 1 [file sj-docx-1-jop-10.1177_02698811261473383.docx]

**The CAMHS Tier 4 Medication Questionnaire**

**PART 2 - Information about the residents of the unit and the medication prescribed on the census date**

**Next Page Previous Page Cancel**

**Clear Submit**

**Project Navigation**

# Introduction

A separate questionnaire should be completed for each person on the facility on the agreed census day

**Section 1: Demographics**

1.1 - Unit Code
1.2 - Unit Type
1.3 - Census Date
1.4 - Gender

/

Select...

Male (including trans man)

16

/

Female (including trans woman)

2021

12

Non-binary

1.5 - Age

Other (not listed)	Not stated (person

asked but declined

to provide a response

1.6 - What is the persons length of stay in the current unit?

Select...

1.7 - Mental Health Act
1.8 - Ethnicity
1.9 - BMI
1.10 - Is the responsible Clinician for this person:
1.11 - Does the person have a Learning Disability?
1.12 - Is the person thought to have Autism?
1.13 - Does the person have Epilepsy?
1.14 - Is there a mental health diagnosis?

Primary Diagnosis

Select...

Informal Detained

Select...

Select...

Substantive? Locum? Yes No

Yes No Yes, is diagnosed

Yes No

Yes No

Select...

Confirmed	Emerging

1.15 - Is there a secondary mental health diagnosis? Secondary Diagnosis

Yes No

Confirmed	Emerging

Select...

1.16 - Other Diagnosis (please specify)
1.17 - Psychotropic Medication Prescribed?

Yes	No

Section 2

Census ID 2021-11

**Section 2: Antipsychotic Prescribing Including Prns**

Antipsychotics (also known as neuroleptics or major tranquillisers) are used to manage psychosis, schizophrenia and bipolar illness.

**Next Page Previous Page Cancel**

**Clear Submit**

**Project Navigation**

**2.1 -**

## Is this person prescribed regular or PRN antipsychotic?

If not proceed to Section 3

Yes	No

## 2.2 - Regular antipsychotic(s) prescribed and dose

Is this person prescribed a regular antipsychotic? Yes No

Tick the box to enable the row, select the drug from picklist and then enter the dose.

## Part 1 - Oral regular

Drug (click in box and select from picklist)

Daily dose (mg)

1st

2nd

3rd

## Part 2 - Depot or long acting injectable antipsychotic

1st

Drug (click in box and select from picklist)

Monthly dose (mg)

## 2.3 - The regular antipsychotic is prescribed for:

 Mood stabilisation

 Psychotic illness or schizophrenia Management of behaviour

 Other (please state here)

## 2.4 - As required prn. antipsychotic prescribed, route and dose

Is the person prescribed an antipsychotic as a prn?	Yes	No

1st

Drug (click in box and select from picklist)

Potential maximum Daily dose (mg)

Route

Oral IM	Either

 2nd

How many doses of prn antipsychotic have been given in the last 2 weeks ?

Select...

## 2.5 - The prn antipsychotic is prescribed for:

 Alleviate Psychotic Phenomena/mood stabilisation Rapid Tranquilisation

 Refusal to accept regular antipsychotics Other (please state here)

## Click on a number to go to a specific section

Previous Page

1

2

3

4

5

6

7

8

9

10

Section 3

**Section 3:**

Anxiolytics and antihistamines are used to manage anxiety. However, the term previously was sedatives or minor tranquillisers. The main group of anxiolytics (the benzodiazepines) are now only recommended for short term use and antidepressants are used for long term anxiety.

**Next Page Previous Page Cancel**

**Clear Submit**

**Project Navigation**

**3.1 -**

## Is this person prescribed regular or PRN benzodiazepine or antihistamine for sedation (not for epilepsy)?

If not proceed to Section 4

Yes	No

**3.2 -**

## Regular benzodiazepine(s) or antihistamines prescribed for sedation and dose

Is this person prescribed a regular benzodiazepine or antihistamine for sedation or anxiety?

Yes	No

Tick the box to enable the row, select the drug from picklist and then enter the dose.

1st

Drug (click in box and select from picklist)

Daily dose (mg)

2nd

**3.3 -**

## As required or prn benzodiazepine and/or antihistamine prescribed for sedation or anxiety, route and dose

Is this person prescribed a prn benzodiazepine or antihistamine?

Yes	No

1st

Drug (click in box and select from picklist)

Potential maximum Daily dose (mg)

Route

Oral IM	Either

2nd

How many doses of prn benzodiazepine or antihistamine doses have been given for sedation or anxiety in the last 2 weeks?

Select...

## Click on a number to go to a specific section

Previous Page

1

2

3

4

5

6

7

8

9

10

Section 4

**Section 4: Antidepressant prescribing**

Antidepressants are used to treat depression and anxiety

**Next Page Previous Page Cancel**

**Clear Submit**

**Project Navigation**

**4.1 -**

## Is this person prescribed regular antidepressant?

If not proceed to Section 5

Yes	No

## 4.2 - Regular antidepressant(s) prescribed and dose

Tick the box to enable the row, select the drug from picklist and then enter the dose.

1st

Drug (click in box and select from picklist)

Daily dose (mg)

2nd

## 4.3 - The regular antidepressant(s) is prescribed for:

 Depression Anxiety

 Obsessive compulsive disorder Other (please state here)

## Click on a number to go to a specific section

Previous Page

1

2

3

4

5

6

7

8

9

10

Section 5

Hypnotics are used to manage sleep. This section includes benzodiazepine hypnotics, Z drugs, melatonin and antihistamines used as hypnotics

**Next Page Previous Page Cancel**

**Clear Submit**

**5.1 -**

## Is this person prescribed regular or PRN hypnotic?

If not proceed to Section 6

Yes	No

## 5.2 - Regular hypnotic(s) prescribed and dose

Is this person prescribed a regular hypnotic? Yes No

Tick the box to enable the row, select the drug from picklist and then enter the dose.

1st

Drug (click in box and select from picklist)

Daily dose (mg)

2nd

## 5.4 - As required or prn hypnotic prescribed and dose

Is a prn hypnotic prescribed?	Yes	No

1st

Drug (click in box and select from picklist)

Daily dose (mg)

 2nd

How many prn hypnotic doses have been given in the last 2 weeks?

Select...

## Click on a number to go to a specific section

Previous Page

1

2

3

4

5

6

7

8

9

10

Section 6

Antiepileptics are also known as anticonvulsants. In addition to treating epilepsy they are useful in managing mood disorders and bipolar illness**.**

**Previous Page Cancel**

**Clear Submit**

**6.1 -**

**6.2 -**

## Is this person prescribed regular antiepileptic?

If not proceed to Section 7

## The antiepileptic is prescribed for

Yes	No

Mood stabilisation An adjunct in schizophrenia

Management of behaviour Epilepsy Other (please state)

## 6.3 - Regular antiepileptic(s) prescribed and dose

Tick the box to enable the row, select the drug from picklist and then enter the dose.

1st

Drug (click in box and select from picklist)

Daily dose (mg)

2nd

3rd

## Click on a number to go to a specific section

Previous Page

1

2

3

4

5

6

7

8

9

10

Section 7

**Section 7: Stimulant or other ADHD**

Stimulants are used to manage attention deficit hyperactivity disorder (ADHD)

**Project Navigation**

- **Next Page**
- **Previous Page**
- **Cancel**
- **Clear**
- **Submit**

**Completion Errors**

**7.1 -**

## Is this person prescribed regular stimulant or other ADHD medication?

If not proceed to Section 8

Yes	No

## 7.2 - Regular ADHD medication(s) prescribed and dose

Tick the box to enable the row, select the drug from picklist and then enter the dose.

1st

Drug (click in box and select from picklist)

Daily dose (mg)

2nd

## Click on a number to go to a specific section

Previous Page

1

2

3

4

5

6

7

8

9

10

Section 8

Antimuscarinic medications are used to manage some side effects of the antipsychotic drugs. This includes movement disorders and hypersalivation.

**Previous Page Cancel**

**Clear Submit**

**8.1 -**

## Is this person prescribed regular antimuscarinic?

If not proceed to Section 9

Yes	No

## 8.2 - Regular antimuscarinic(s) prescribed and dose

Tick the box to enable the row, select the drug from picklist and then enter the dose.

1st

Drug (click in box and select from picklist)

Select...

Daily dose (mg)

## Click on a number to go to a specific section

Previous Page

1

2

3

4

5

6

7

8

9

10

Section 9

**9.1 -**

**Next Page Previous Page Cancel**

**Clear Submit**

**Project Navigation**

**Section 9: Other psychotropic medication prescribed (eg lithium, herbal sedatives)**

## Is this person prescribed another regular psychotropic medication not included before?

If not proceed to Section 10

Yes	No

## 9.2 - Regular other psychotropic medication prescribed and dose

Daily dose (mg)

Is the person prescribed Lithium?

Yes

Tick the box to enable the row, enter the drug name and then enter the dose.

1st

Drug (Type name in the box)

Daily dose (mg)

2nd

## Click on a number to go to a specific section

Previous Page

1

2

3

4

5

6

7

8

9

10

Section 10

For definitions please refer to the Appendix

**Next Page Previous Page Cancel**

**Clear Submit**

**Project Navigation**

Appendix

**Section 10: Appropriate or inappropriate polypharmacy**

**10.0 -**

## Is this person prescribed more than one psychotropic medication?

Yes	No

## 10.1 - Total polypharmacy. How many psychotropic medications are prescribed

**for this person?**

Regular psychotropic medication only

Regular and prn psychotropic medications prescribed

0

0

## 10.2 - Same class polypharmacy.

Does the multiple psychotropic same class prescribing meet the criteria for appropriate or inappropriate polypharmacy?

Complete the table below for this person (Guidance - complete together with the consultant)

**Medication category**

**Prescribed for this person**

**Choose whether you think this is appropriate or inappropriate polypharmacy or you are unsure**

More than 1 antipsychotic

Regular and prn antipsychotic

More than one benzodiazepine or antihistamine for sedation including prn

More than 1 antidepressant

More than one hypnotic including prn

(0 antipsychotic(s) selected)

(0 regular and prn's selected)

(0 benzodiazepine(s), or and prn(s) selected)

(0 antidepressant(s) selected)

(0 hypnotic(s), or and prn(s) selected)

## Click on a number to go to a specific section

1

2

3

4

5

6

7

8

9

10

Previous Page

Appendix

Next Page

**Section 10: Appropriate or inappropriate polypharmacy - cont.**

10.3 **-**

**Next Page Previous Page Cancel**

**Clear Submit**

**Project Navigation**

## Multiclass polypharmacy - Is the combination of psychotropic medications this person receives regularly appropriate or inappropriate polypharmacy?

List of regular psychotropic medication classes that this person receives

Choose whether you think this is appropriate or inappropriate polypharmacy or you are unsure

Select...

10.4 **-**

## Multiclass polypharmacy - Is the combination of psychotropic medications this person receives regularly and potentially through prn appropriate or inappropriate polypharmacy?

List of regular and prn psychotropic medication classes that this person receives

Choose whether you think this is appropriate or inappropriate polypharmacy or you are unsure

Select...

## 10.5 - Potential to reduce the psychotropic load

Select...

# Thank you for your assistance.

**Please press 'Submit' below and then move on to the next person.**

Submit Audit

## Click on a number to go to a specific section

1

2

3

4

5

6

7

8

9

10

Previous Page

Appendix

**Appendix**

## Definition of polypharmacy

**Project Navigation**

- **Next Page**
- **Previous Page**
- **Cancel**
- **Clear**
- **Submit**

**Completion Errors**

(Duerden M, Avery T, Payne R. (2013) Polypharmacy and Medicines Optimisation. Making it safe and sound. The Kings Fund

[PDF is available here](https://www.kingsfund.org.uk/sites/default/files/field/field_publication_file/polypharmacy-and-medicines-optimisation-kingsfund-nov13.pdf)

- Appropriate polypharmacy is defined as prescribing for an individual for complex conditions or for multiple conditions in circumstances where medicines use has been optimised and where the medicines are prescribed according to best evidence.
- Problematic polypharmacy is defined as the prescribing of multiple medications inappropriately, or where the intended benefit of the medication is not realised.

## Categories of polypharmacy

Due to the increasing prevalence and complexity in psychiatric polypharmacy, it is categorised as follows by the National Association of State Mental Health Programme Directors (NASMHPD) (NASMHPD Technical Report, 2001[32]):

- Same-Class Polypharmacy refers to the use of more than one medication from the same class (e.g. use of two selective serotonin reuptake inhibitors in a case of depression).
- Multi-Class Polypharmacy is the use of full therapeutic doses of more than one medication from different classes for the same symptom cluster (e.g. use of valproate along with an atypical antipsychotic, such as olanzapine, for treatment of mania).
- Use of one medication to treat the side effects of another medication from a different class, is described as Adjunctive Polypharmacy (e.g. using trazodone for insomnia caused by bupropion).
- Augmentation Polypharmacy refers to the use of one medication at a lower than normal dose along with another medication from a different class in full therapeutic dose for the same symptom cluster (e.g. addition of low dose haloperidol in a patient responding partially to risperidone); or the addition of a medication that would not be used alone for the same symptom cluster (e.g. augmentation of antidepressants with lithium or thyroid hormone).

Total Polypharmacy is the total count of medications used in a patient, or total drug load.

Return to Section 10

# Thank you for your assistance.

**Project Navigation**

- **Next Page**
- **Previous Page**
- **Cancel**
- **Clear**
- **Submit**

**Completion Errors**

**As this person is not prescribed any psychotropic medication please press 'Submit' below and then move on to the next person.**

Submit Audit

# Thank you for your assistance.

**Project Navigation**

- **Next Page**
- **Previous Page**
- **Cancel**
- **Clear**
- **Submit**

**Completion Errors**

**As this person is only prescribed one psychotropic medication please press 'Submit' below and then move on to the next person.**

Submit Audit
