# Supplementary material for: Psychotropic medication prescribing to children and young people mental health inpatient services: An English national study
Source: J Psychopharmacol. 2026 Aug 12;40(10):1594–607. doi: 10.1177/02698811261473383 (PMC13683887; doi:10.1177/02698811261473383)
Supplement: Supplementary file 2 [file sj-docx-2-jop-10.1177_02698811261473383.docx]

**TELL US ABOUT YOUR MEDICATION**

**--.a::,c:D.,e::�**

There is a piece of work that the NHS are leading on which is about people having the right medication and treatment for their mental health.

A lot of children and young people who are autistic, have a learning disability or have mental health needs, take medication.

It is very important that you get the right medication, at the right time and for the right reasons.

Please answer these questions to tell us what you know and how you feel abOL'� .,,..., .. """':dication.

�--§

**f	1**

### It's your choice!

1. Only answer the questions you want to.
2. If you want to talk to someone about these questions, ask someone you trust for help.
3. Please read the questionnaire information page before you answer the questions.

### 1 - Do you take medication?

D Yes	□ No	D I don't know

### 2 - Do you know what medication you take?

D Yes	D No	D I don't know

### 3 - Does your medication help you?

D Yes	D No	D I don't know

4- Why do you take your medication?

### 5 - Is there a member of staff you feel you can ask questions about your medication?

D Yes	□ No	D I don't know

6- What would make it easier for you to ask staff questions about your medication?

### 7 - Do you feel your medication stops you doing the things you enjoy?

D Yes	□ No

D Sometimes	D Don't Know

Write in this box anything else you want to tell us about this

### 8 - Do you have any worries about the medication you are on?

D Yes	D No	D I don't know If yes, what worries do you have about your medication?

### 9 - Medication can be one way to help you stay healthy and well.

What other things would you like to do to

stay healthy and well?

- **Please tick how you get information**

**10**

**ab th**

ID

**out your medication; you can tick more**

**an one box if you needed.**

Information leaflet

□ A doctor or nurse talks to me about it

□ My family talk to me about it

- I already have information
- I don't get information
- I don't understand the information I get

□

Other: If you get information about your

medication in other ways, write it in the box below

**11** - **If you get information about your medication, do you find it helpful?**

- Oves

I,

D

Sometimes

D Don't Know

What would make the information more helpful?

# Thank you for all your help

If you have any worries about your medication, please tell someone you trust.

All images copyright ©David Gill 2021

## Questionaire About Medication Information Page

#### What is this questionnaire?

A questionnaire is a list of questions to find out what you think about something. This questionnaire is about psychotropic mental health medication for example, medicine and tablets.

#### What is medication for mental health?

Medications for mental health can be used to treat things like for example, anxiety, attention deficit hyperactivity disorder (ADHD), depression, sleep problems, epilepsy and psychosis.

#### Who is this questionnaire from?

The NHS Quality Improvement team want to make sure that children and young people are only taking the right medication, at the right time and for the right reasons. To help do this, please can you answer the questions on the questionnaire. This will help us to understand how you feel about your medication.

#### Who is it for?

This questionnaire is for any young person aged between 13 and 18 years of age who is taking medication for their mental health or wellbeing, while on a ward in a mental health inpatient hospital.

## Questionaire About Medication Information Page - continued

#### Do I have to fill in this questionnaire?

It's completely up to you. You don't have to fill in this questionnaire if you don't want to.

You can read the questionnaire before you decide whether you want to fill it in.

You can leave out any questions you don't want to answer.

You don't have to fill it in one go. You can take your time and come back to it later.

You can ask for help and support with filling this questionnaire.

#### Who can I ask for support?

You can ask anyone you trust to help you fill in this questionnaire. Some people you might want to ask could be:

Your Family

A support worker A close friend

An independent advocate A member of staff Anyone else you trust

#### What will happen to the information I am giving?

We are asking lots of people these questions. We will use the answers to help improve care and treatment for children and young people. We won't ask you anything about your name, date of birth and address and members of staff from your ward will not be able to see your responses. Any names of people that are entered will be deleted from the data collected. The only people that will see your answers are from the NHS Quality Improvement team.
