# Supplementary material for: Psychotropic medication prescribing to children and young people mental health inpatient services: An English national study
Source: J Psychopharmacol. 2026 Aug 12;40(10):1594–607. doi: 10.1177/02698811261473383 (PMC13683887; doi:10.1177/02698811261473383)
Supplement: Supplementary file 3 [file sj-docx-3-jop-10.1177_02698811261473383.docx]

# Tell us about your child’s medication

A lot of children and young people who have mental health needs, are autistic, or have a learning disability take medication for their mental health. **Psychotropic medication** is any drug capable of affecting the mind, emotions and behaviour.

## Who is this questionnaire from?

The NHS Quality Improvement Taskforce team want to make sure children and young people are only taking the right medication, at the right time and for the right reasons.

To help to do this, please can you answer these questions to tell us what you know and your views about **the psychotropic medication** prescribed for your child.

Parents can fill the questionnaire in together or separately.

## Who is it for?

This questionnaire is for any parent/family carer of a young person aged between 13 and 18 years of age who is taking medication for their mental health or wellbeing, while on a ward in a mental health inpatient hospital.

## What will happen to the information I am giving?

We are asking lots of people these questions. We won’t ask you anything about you or your child’s name, date of birth or address. This means that people will not be able to identify you or them from your answers. Any names of people that are entered by people filling in the questionnaire will be deleted from the data collected. As this questionnaire is anonymous,

we cannot follow up on individual concerns. There is information at the end of the questionnaire about how to raise concerns about your child’s medication.

The information that you provide will feed into a national report that will support further quality improvement projects. It will also be fed back as part of an overall results dashboard to your child’s service (anonymised) so that it can be used to make improvements.

**Examples of psychotropic medication** to treat:

- Depression and anxiety – antidepressants such as fluoxetine, citalopram, venlafaxine
- Anxiety – benzodiazepines such as lorazepam and diazepam, and antihistamines such as promethazine
- Psychosis and schizophrenia – antipsychotics such as risperidone, olanzapine, quetiapine, aripiprazole
- Mood and bipolar illness – antiepileptics such as sodium valproate
- Attention deficit hyperactivity disorder (ADHD) – stimulants such as methylphenidate
- Sleep problems – hypnotics such as melatonin, z drugs like zopiclone, antihistamines and benzodiazepines such as temazepam.

1. Does your child take psychotropic medication? This survey is only relevant if your child takes psychotropic medication.

Yes No I don’t know

2. Do you know what medication they take? I know **all** their medication

I know **some of** their medication

No I am not sure

3. In your opinion, why is your child prescribed psychotropic medication?
4. Do you feel you have enough information to ask informed questions about the use of psychotropic medication?

Yes No	I don’t know

5. Is there a member of staff who you feel you can ask questions about your child’s medication?

Yes No

If yes, who do you ask and what did you need to ask?

6. What would make is easier for you to ask staff?
7. Do you feel the psychotropic medication helps your child? I feel it **all** helps my child

I feel **some of** it helps my child No, it doesn’t help my child

I don’t know if it helps my child

Use this space to tell us more about which medications you feel help or don’t help your child

8. Does psychotropic medication impact your child’s wellbeing and motivation? For example, does it stop them doing the things they enjoy?

Yes No

Sometimes Don’t know Write in this box anything else you want to tell us about this

9. Do you have any worries about the psychotropic medication your child is taking?

Yes No

If yes, what worries do you have about their medication? For example the amount, type or frequency of medication or the information you were given about it?

10a. Are you involved in decisions about your child’s psychotropic medication?

Yes No

10b. If you don’t agree with the use of medication, do you feel you can discuss this with staff?

Yes No

10c. Do you feel your views about psychotropic medication are listened to and taken into account?

Yes No I don’t discuss decisions Would you like to tell us more about this?

11a. Was psychotropic medication the only intervention offered to your child?

Yes No I don’t know

11b. What alternatives to psychotropic medication were you offered or would have been useful? What other things do you feel your child would benefit from to stay healthy and well?

12. Please tick how you get information about your child’s psychotropic medication; you can tick more than one box if you need.

Information leaflet

My child’s doctor or nurse talks to me about it My child talks to me about it

The internet

I don’t get information

Other: if you get information about medication in other ways, write it in the box below

13. If you get information about your child’s medication, do you find it helpful?

Yes No

Sometimes I don’t get information What would make the information more helpful?

## If you have any worries about your child’s medication and you need support or guidance the following people can help:

- Your child’s named nurse
- Your child’s Responsible Clinician (if you don’t know who the Responsible Clinician is for your child, ask their named nurse)
- Your child’s advocate
- Some wards have access to a Family Ambassador who can help you raise concerns with your child’s multi-disciplinary team (MDT). The ward manager can tell you whether there is a Family Ambassador in your area.
- The NHS STAMP website has more information and signposts to other people who can help. <https://www.england.nhs.uk/learning-disabilities/improving-health/stamp/>
- The Challenging Behaviour Foundation website has a Medication Pathway resource which may be helpful: [https://medication.challengingbehaviour.org.uk/pathway/sc](https://medication.challengingbehaviour.org.uk/pathway/scenarios-section-c/) [enarios-section-c/](https://medication.challengingbehaviour.org.uk/pathway/scenarios-section-c/)

**Thank you for all your help**
