# Supplementary material for: Psychotropic medication prescribing to children and young people mental health inpatient services: An English national study
Source: J Psychopharmacol. 2026 Aug 12;40(10):1594–607. doi: 10.1177/02698811261473383 (PMC13683887; doi:10.1177/02698811261473383)
Supplement: Supplementary file 4 [file sj-docx-4-jop-10.1177_02698811261473383.docx]

**Title and Abstract**

**Title**: Indicate that the manuscript concerns an initiative to improve healthcare (broadly defined to include the quality, safety, effectiveness, patient-centeredness, timeliness, cost, efficiency, and equity of healthcare) page 1

**Abstract**:

a. Provide adequate information to aid in searching and indexing
b. Summarize all key information from various sections of the text using the abstract format of the intended publication or a structured summary such as: background, local [problem](http://squire-statement.org/index.cfm?fuseaction=page.viewpage&pageid=485#Problem), methods, interventions, results, conclusions Page 3

**Introduction**

**Problem**: Nature and significance of the local [problem](http://squire-statement.org/index.cfm?fuseaction=page.viewpage&pageid=485#Problem)

Page 5

**Available Knowledge**: Summary of what is currently known about the [problem](http://squire-statement.org/index.cfm?fuseaction=page.viewpage&pageid=485#Problem), including relevant previous studies

Page 5-6

**Rationale**: Informal or formal frameworks, models, concepts, and/or [theories](http://squire-statement.org/index.cfm?fuseaction=page.viewpage&pageid=485#Theory) used to explain the [problem](http://squire-statement.org/index.cfm?fuseaction=page.viewpage&pageid=485#Problem), any reasons or [assumptions](http://squire.citysoft.org/index.cfm?fuseaction=page.viewPage&pageID=485&nodeID=1#assumptions) that were used to develop the [intervention(s),](http://squire-statement.org/index.cfm?fuseaction=page.viewpage&pageid=485#Interventions) and reasons why the [intervention(s)](http://squire-statement.org/index.cfm?fuseaction=page.viewpage&pageid=485#Interventions) was expected to work

Page 6

**Specific Aims**: Purpose of the project and this report

Page 6

**Methods**

**Context**: Contextual elements considered important at the outset of introducing the intervention(s)

Page 6-9

**Intervention(s):**

a. Description of the intervention(s) in sufficient detail that others could reproduce it
b. Specifics of the team involved in the work

Page 6-9

**Study of the Intervention(s):**

a. Approach chosen for assessing the impact of the intervention(s)
b. Approach used to establish whether the observed outcomes were due to the intervention(s)

Page 6-9

**Measures**:

a. Measures chosen for studying processes and outcomes of the intervention(s), including rationale for choosing them, their operational definitions, and their validity and reliability
b. Description of the approach to the ongoing assessment of contextual elements that contributed to the success, failure, efficiency, and cost
c. Methods employed for assessing completeness and accuracy of data

Page 6-9

**Analysis**:

a. Qualitative and quantitative methods used to draw inferences from the data
b. Methods for understanding variation within the data, including the effects of time as a variable

Page 6 -9

**Ethical Considerations**: [Ethical aspects](http://squire-statement.org/index.cfm?fuseaction=page.viewpage&pageid=485#Ethical_aspects) of implementing and studying the [intervention(s)](http://squire-statement.org/index.cfm?fuseaction=page.viewpage&pageid=485#Interventions) and how they were addressed, including, but not limited to, formal ethics review and potential conflict(s) of interest

Page 9

**Results**

**Result**:

a. Initial steps of the intervention(s) and their evolution over time (e.g., time-line diagram, flow chart, or table), including modifications made to the intervention during the project
b. Details of the process measures and outcome
c. Contextual elements that interacted with the intervention(s)
d. Observed associations between outcomes, interventions, and relevant contextual elements
e. Unintended consequences such as unexpected benefits, problems, failures, or costs associated with the intervention(s).
f. Details about missing data

Page 9 to 11 table 1-6

**Discussion**

**Summary**:

a. Key findings, including relevance to the rationale and specific aims
b. Particular strengths of the project

Page 12-13

**Interpretation**:

a. Nature of the association between the intervention(s) and the outcomes
b. Comparison of results with findings from other publications
c. Impact of the project on people and systems
d. Reasons for any differences between observed and anticipated outcomes, including the influence of context
e. Costs and strategic trade-offs, including opportunity costs

Page 12-13

**Limitations**:

a. Limits to the generalizability of the work
b. Factors that might have limited internal validity such as confounding, bias, or imprecision in the design, methods, measurement, or analysis
c. Efforts made to minimize and adjust for limitations

Page 13

**Conclusions**:

a. Usefulness of the work
b. Sustainability
c. Potential for spread to other contexts
d. Implications for practice and for further study in the field
e. Suggested next steps

Page 13

**Other**

**Funding**: Sources of funding that supported this work. Role, if any, of the funding

organization in the design, implementation, interpretation, and reporting

Title page
