# Supplementary material for: Psychotropic medication prescribing to children and young people mental health inpatient services: An English national study
Source: J Psychopharmacol. 2026 Aug 12;40(10):1594–607. doi: 10.1177/02698811261473383 (PMC13683887; doi:10.1177/02698811261473383)
Supplement: Supplementary file 6 [file sj-docx-6-jop-10.1177_02698811261473383.docx]

Classification: Official-Sensitive

| To: Whom it may concern | NHS England Wellington House  133-155 Waterloo Road  London SE1 8UG  21 January 2025 |
| --- | --- |

Dear Sir/Madam

This is confirmation that -The Psychotropic Medication in Children and Young People’s (CYP) Mental Health In-patient Services – A Quality Improvement Programme is a NHS England programme. NHS England is the data processor of the Quality Improvement Programme and has responsibility for its governance.

In autumn 2019, a National Quality Improvement Taskforce (NQIT) was established by NHS England to improve current specialist children and young people’s (CYP) inpatient mental health, autism and learning disability services in England. This psychotropic medication improvement programme was developed and overseen at NHS England as a part of that taskforce programme.

This improvement programme has provided an important opportunity for providers of inpatient mental health services for children and young people across England to critically evaluate and compare their use of psychotropic medication. It has also provided an opportunity to hear from young people, parent/carers, and clinicians about the current challenges in psychotropic use in this setting including information sharing, polypharmacy and the concerns about the risks of psychotropic medication use in this population.

NHS England has granted permission to the authors to proceed to publish their findings in peer review journals.

Leanne Gelder

**Head of Quality Transformation (Reducing Restrictive Practice and Culture)National Mental Health, Learning Disability and Autism Quality Transformation Programme, NHS England**

Copyright © NHS England 2025	2
